# Nociceptive Local Field Potentials Recorded from the Human Insula Are Not Specific for Nociception

**DOI:** 10.1371/journal.pbio.1002345

**Published:** 2016-01-06

**Authors:** Giulia Liberati, Anne Klöcker, Marta M. Safronova, Susana Ferrão Santos, Jose-Geraldo Ribeiro Vaz, Christian Raftopoulos, André Mouraux

**Affiliations:** 1 Institute of Neuroscience, Université catholique de Louvain, Brussels, Belgium; 2 Department of Radiology, Neuroradiology Unit, Saint-Luc University Hospital, Brussels, Belgium; 3 Department of Neurology, Saint-Luc University Hospital, Brussels, Belgium; 4 Department of Neurosurgery, Saint-Luc University Hospital, Brussels, Belgium; Northwestern University, UNITED STATES

## Abstract

The insula, particularly its posterior portion, is often regarded as a primary cortex for pain. However, this interpretation is largely based on reverse inference, and a specific involvement of the insula in pain has never been demonstrated. Taking advantage of the high spatiotemporal resolution of direct intracerebral recordings, we investigated whether the human insula exhibits local field potentials (LFPs) specific for pain. Forty-seven insular sites were investigated. Participants received brief stimuli belonging to four different modalities (nociceptive, vibrotactile, auditory, and visual). Both nociceptive stimuli and non-nociceptive vibrotactile, auditory, and visual stimuli elicited consistent LFPs in the posterior and anterior insula, with matching spatial distributions. Furthermore, a blind source separation procedure showed that nociceptive LFPs are largely explained by multimodal neural activity also contributing to non-nociceptive LFPs. By revealing that LFPs elicited by nociceptive stimuli reflect activity unrelated to nociception and pain, our results confute the widespread assumption that these brain responses are a signature for pain perception and its modulation.

## Abstract 2

Local field potentials elicited in the human insular cortex by painful stimuli reflect cortical activity that is unrelated to pain perception and so cannot be used as an objective measure of pain.

## Author Summary

A widely accepted notion is that the insula, especially its posterior portion, plays a specific role in the perception of pain. This has led a number of researchers to consider activity recorded from this so-called “ouch zone” as an objective correlate of pain perception. We provide compelling evidence to the contrary. Using direct intracerebral recordings, we demonstrate that painful and nonpainful stimuli elicit very similar responses throughout the human insula. This observation argues against the notion that these responses reflect the brain activity through which pain emerges from nociception in the human brain. These findings have implications for basic theories, as well as for the development of diagnostic tests and the identification of therapeutic targets for the treatment of chronic pain. They question the use of these insular responses to assess the effects of pharmacological treatment or to assess pain in patients unable to communicate. Furthermore, they have legal implications, as they contradict the proposal that these responses could be used to determine unequivocally whether plaintiffs are truly experiencing the pain for which they are seeking redress. Finally, they undermine the rationale for neurosurgical procedures aiming at alleviating pain by targeting the posterior insula.

## Introduction

The human insula, in particular the region encompassing the dorsal posterior insula and the adjacent parietal operculum, is generally believed to play a specific role in the perception of pain. There are several reasons behind this belief. First, the insula is an important cortical target for nociceptive inputs ascending the spinothalamic tract [1]. Second, direct electrical stimulation of the human insula, as well as focal epileptic seizures in this region, may trigger an acute experience of pain [2–4]. Third, lesions of the insula may lead to a selective impairment of the ability to perceive nociceptive stimuli, as well as central pain [5]. Fourth, depth recordings in humans have shown that nociceptive stimuli elicit robust LFPs in this region, considered to reflect early stages of cortical processing specifically related to the perception of pain [6–9]. Fifth, electroencephalography (EEG), positron emission tomography (PET), and functional magnetic resonance imaging (fMRI) studies have shown consistently that the insula is activated by stimuli perceived as painful [10–16]. Finally, several studies have shown a significant correlation between the magnitude of the responses recorded in the insula and the intensity of perceived pain [15,17–20]. In particular, Segerdahl et al. [18] recently demonstrated a significant correlation between long-lasting changes in absolute cerebral blood flow (CBF) in the dorsal posterior insula and the intensity of perceived ongoing pain. All these observations provide support for a specific involvement of the insula in pain perception.

Yet, this conclusion is challenged by several counterarguments or differing findings. Because they imply necessity and sufficiency, lesion studies and focal seizure cases could be expected to provide unequivocal evidence for a specific involvement of the insula in pain perception. However, the notion that pain constitutes a common ictal symptom associated with insular discharge comes from observations performed in only a few patients [3,4]. Furthermore, direct electrical stimulation of the insula in these patients appears to predominantly elicit nonpainful paresthesiae or warm sensations, especially when the stimulated area is not epileptogenic [2,21]. Finally, reports of insular lesions leading to impaired pain perception have been recently questioned by a study of 24 patients with stroke lesions involving the insula, in which no measurable change in pain thresholds could be objectified using quantitative sensory testing [22]. Most importantly, the assumption that the responses triggered in the insula by nociceptive stimuli are specific for pain is based on reverse inference, and the likelihood of this inference being correct depends on the exclusivity of the relationship between these responses and the experience of pain. In other words, to test whether these responses are specific for pain, one must not only demonstrate that stimuli perceived as painful elicit responses in the insula but also that these responses are elicited if and only if the stimulus is painful.

Alongside the assumed pivotal role of the insula in pain perception, it is also widely acknowledged that the insula is involved in the processing of a range of non-nociceptive sensory inputs and that the insula contributes to a large number of cognitive, affective, interoceptive, and homeostatic functions, independently of sensory modality [23–30]. This is not surprising given the heterogeneous cytoarchitecture of the insula and its anatomical connections with a wide array of brain regions [31–36]. Therefore, at least part of the activity recorded in the insula while perceiving pain could reflect cognitive processes that are not specifically related to the pain experience, such as processes involved in orienting attention towards salient stimuli or processes involved in the production of homeostatic responses.

**Fig 1:**
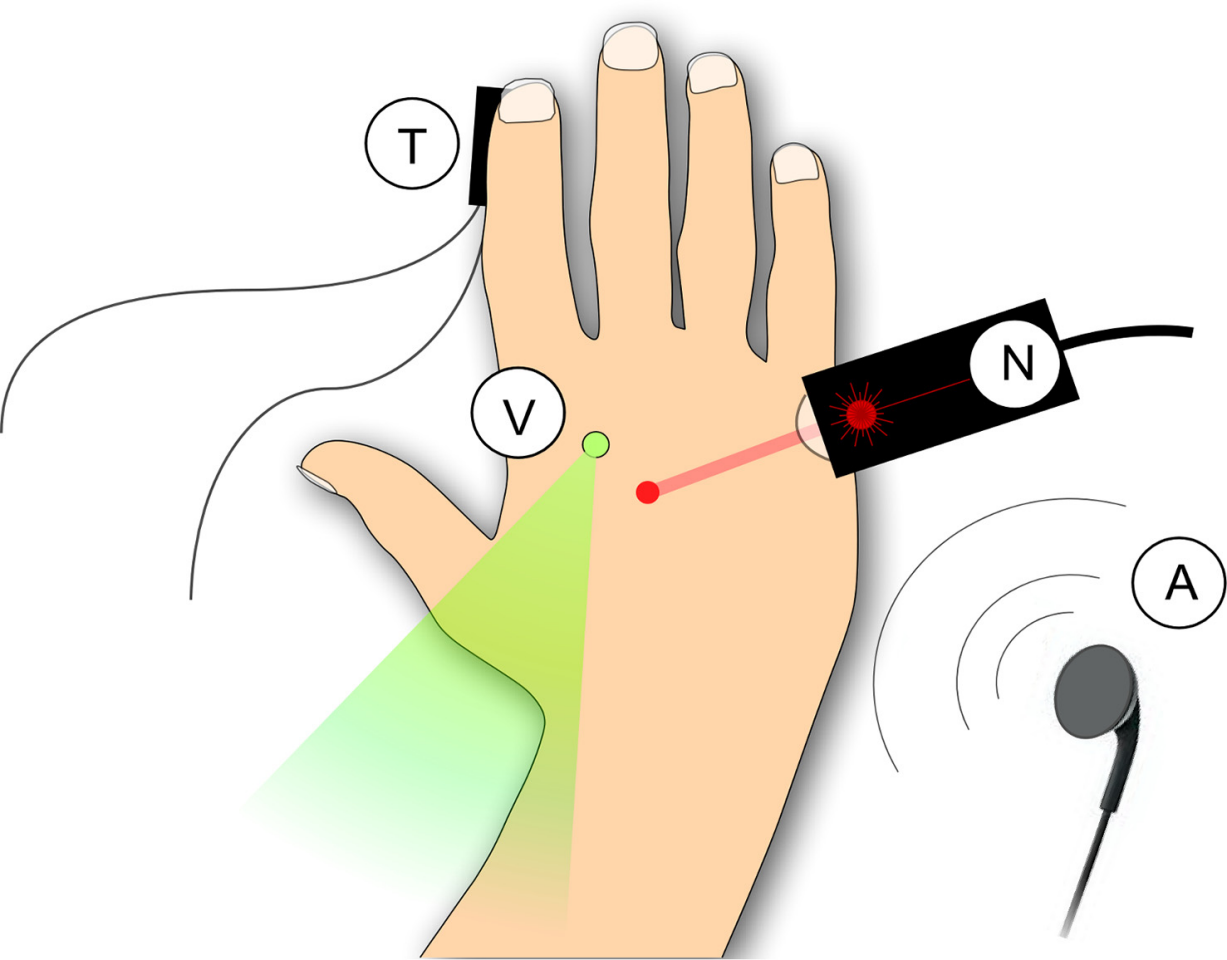
Experimental procedure. Nociceptive stimuli (N) were brief pulses of radiant heat applied to the hand dorsum using a temperature-controlled CO_2_ laser. This ensured that the elicited brain responses were exclusively related to the activation of heat-sensitive nociceptors. Tactile stimuli (T) were short-lasting mechanical vibrations delivered to the index fingertip, so as to selectively activate low-threshold mechanoreceptors of the medial lemniscal system. Auditory stimuli (A) were loud, lateralized short-lasting tones delivered through earphones. Visual stimuli (V) were brief, bright, and punctate flashes of light delivered using a light-emitting diode (LED) placed on the hand dorsum. The different stimuli were presented in blocks, using a long-lasting and variable interstimulus interval (5–10 s), so as to maximize their salience.

The aim of the present study was to address this outstanding question, i.e., to examine whether the insula exhibits responses specific for nociception and the perception of pain. For this purpose, we took advantage of the high spatiotemporal resolution of depth intracerebral EEG recordings performed in humans for the evaluation of refractory focal epilepsy [37]. Using a very straightforward experimental paradigm (see Methods section), we compared the LFPs triggered by nociceptive stimuli eliciting a perception of pain to the LFPs triggered by non-nociceptive and nonpainful vibrotactile, auditory, and visual stimuli (Fig 1). We found that all four types of stimuli elicit highly similar LFPs in both the anterior and posterior portions of the insula. This indicates that, unlike previously thought, the greater part of the insular response to stimuli perceived as painful reflects multimodal activity that is entirely unspecific to pain.

## Results

### Both Nociceptive and Non-nociceptive Stimuli Elicit Consistent LFPs in the Insula

Recordings were obtained from a total of 72 contacts (47 localized in the insula: 22 in the posterior insula, 25 in the anterior insula, and 25 at locations adjacent to the insula) in six patients (four patients with one electrode in the left insula, one patient with one electrode in the right insula, and one patient with electrodes in both the left and right insula). The anterior insula was identified as the region encompassing the short insular gyri (anterior, middle, and posterior), the pole of the insula, and the transverse insular gyrus. The posterior insula was identified as the region composed of the anterior and posterior long insular gyri [38].

Although nociceptive stimuli elicited a clear burning/pricking sensation that was systematically qualified as painful, all stimuli were perceived as equally intense (the average ratings of intensity of perception were not significantly different across sensory modalities; F = .595; *p* = .628).

**Fig 2:**
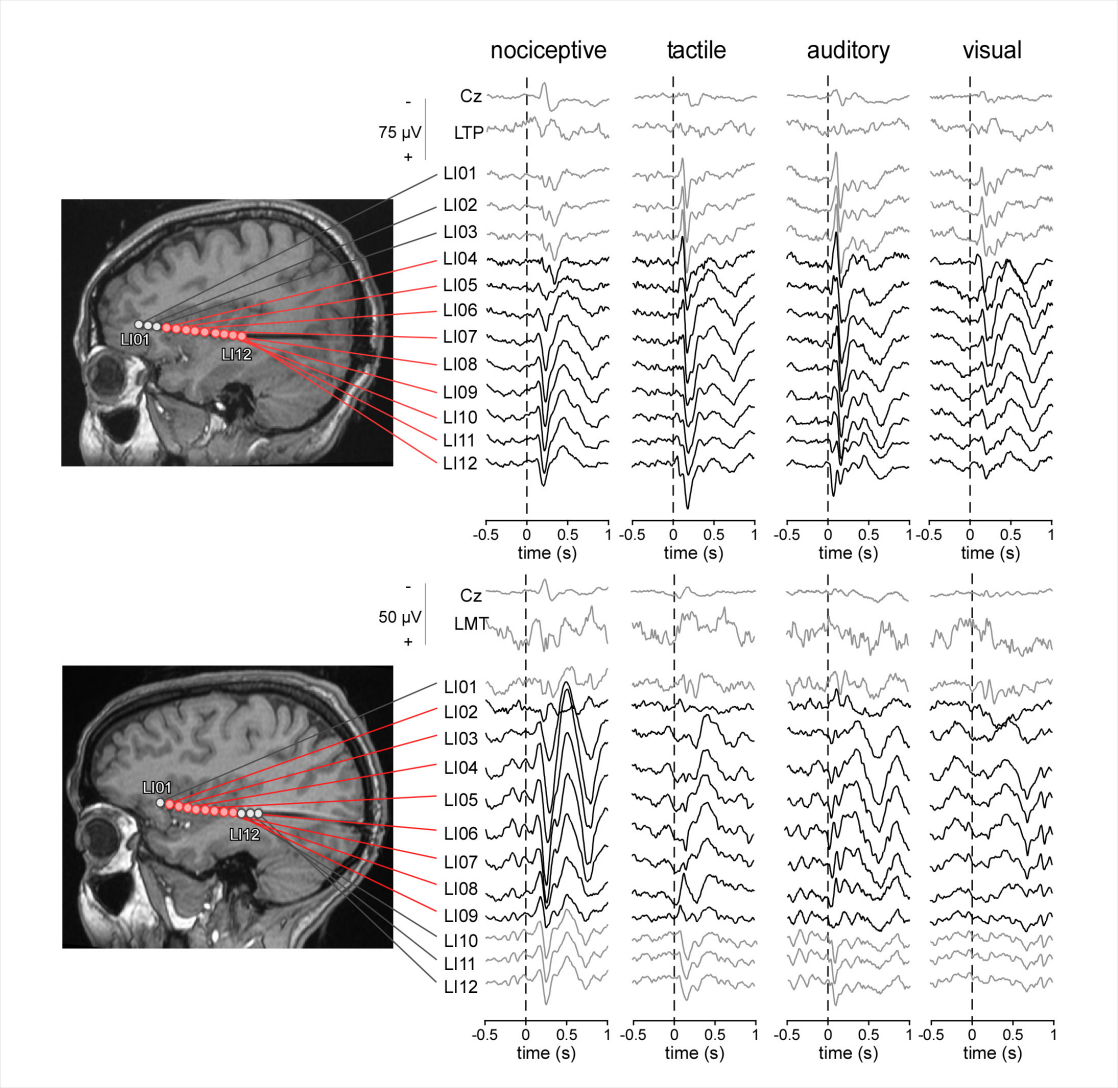
LFPs elicited by nociceptive, tactile, auditory and visual stimuli in the contralateral anterior and posterior insula of two representative subjects. All four types of stimuli elicited responses at the same electrode contacts, both in the anterior insula and in the posterior insula (electrode contacts shown in red; reference electrode: A1A2). The greater part of the LFP appeared as a large biphasic wave. The evoked potentials recorded from the scalp vertex (Cz) and from intracerebral contacts located in the left temporoparietal cortex (LTP) and left mesial temporal cortex (LMT) are also shown for comparison. doi:10.17605/OSF.IO/4R7PM.

In all patients, all four types of stimuli elicited clear LFPs at anterior and posterior insular contacts, appearing as large biphasic waves. The waveforms obtained at each insular contact of two representative subjects are shown in Fig 2. The waveforms obtained in all the other participants are shown in S1 Fig.

The latency and absolute amplitude of each of the two peaks were measured at each insular electrode contact and compared using a linear mixed models (LMM) analysis with “modality” (nociceptive, vibrotactile, auditory, and visual), “contact location” (anterior and posterior insular contacts) and “side” (stimuli delivered to the ipsilateral or contralateral side relative to the explored insular cortex) as fixed factors and “subject” as a contextual variable.

On average, the latencies of the first peak (nociceptive: 184 ± 50 ms; vibrotactile: 113 ± 40 ms; auditory: 89 ± 23 ms; and visual: 140 ± 36 ms) and of the second peak (nociceptive: 296 ± 78 ms; vibrotactile: 205 ± 74 ms; auditory: 161 ± 31 ms; and visual: 216 ± 69 ms) were significantly different across modalities (main effect of “modality”; first peak: F = 125.25, *p* < .001; second peak: F = 95.86, *p* < .001). Post-hoc comparisons showed that the average latency of the responses to nociceptive stimuli was significantly greater than the average latency of the responses to auditory (first peak: *p* < .001; second peak: *p* < .001), vibrotactile (first peak: *p* < .001; second peak: *p* < .001), and visual (first peak: *p* < .001; second peak: *p* < .001) stimuli. These across-modality differences in latency can be explained by the difference in the time required for the sensory afferent volleys to reach the cortex [39,40]. In particular, the greater latency of the responses elicited by nociceptive stimulation as compared to vibrotactile stimulation (latency difference of the first peak: 71 ± 90 ms; latency difference of the second peak: 91 ± 152 ms) can be explained by the fact that small-diameter A-delta fibers conveying nociceptive input have a slower conduction velocity than large-diameter A-beta fibers conveying vibrotactile input.

The latencies of the responses to stimuli delivered to the contralateral side (first peak: 123 ± 47 ms; second peak: 204 ± 60 ms) and ipsilateral side (first peak: 139 ± 56 ms; second peak: 236 ± 97 ms) relative to the explored insula were significantly different (main effect of “side”; first peak: F = 21.16, *p* < .001; second peak: F = 33.21, *p* < .001). Independently of the modality of the eliciting stimulus, the responses elicited by stimulation of the ipsilateral side were, on average, slightly delayed as compared to the responses elicited by stimulation of the contralateral side. This is compatible with previous reports also showing a small latency difference between insular LFPs elicited by nociceptive stimuli delivered to the ipsilateral versus contralateral hand [41]. In contrast, there was no significant effect of the factor “contact location” (first peak: F = 0.32, *p* = .569; second peak: F = 0.64, *p* = .424).

The amplitudes of the first peak (nociceptive: 19 ± 16 μV; vibrotactile: 13 ± 10 μV; auditory: 24 ± 15 μV; and visual: 11 ± 9 μV) and the amplitudes of the second peak (nociceptive: 31 ± 20 μV; vibrotactile: 32 ± 18 μV; auditory: 27 ± 19 μV; and visual: 24 ± 19 μV) were significantly different across modalities (main effect of “modality”: first peak: F = 27.49, *p* < .001; second peak: F = 5.34, *p* = .001). Post-hoc comparisons showed that the amplitude of the first peak was significantly greater for the responses to auditory stimulation as compared to nociceptive (*p* = .010), vibrotactile (*p* < .001), and visual (*p* < .001) stimulation and that the amplitude of the second peak was significantly smaller for the responses to visual stimulation as compared to nociceptive (*p* = .009) and vibrotactile (*p* = .004) stimulation.

**Fig 3:**
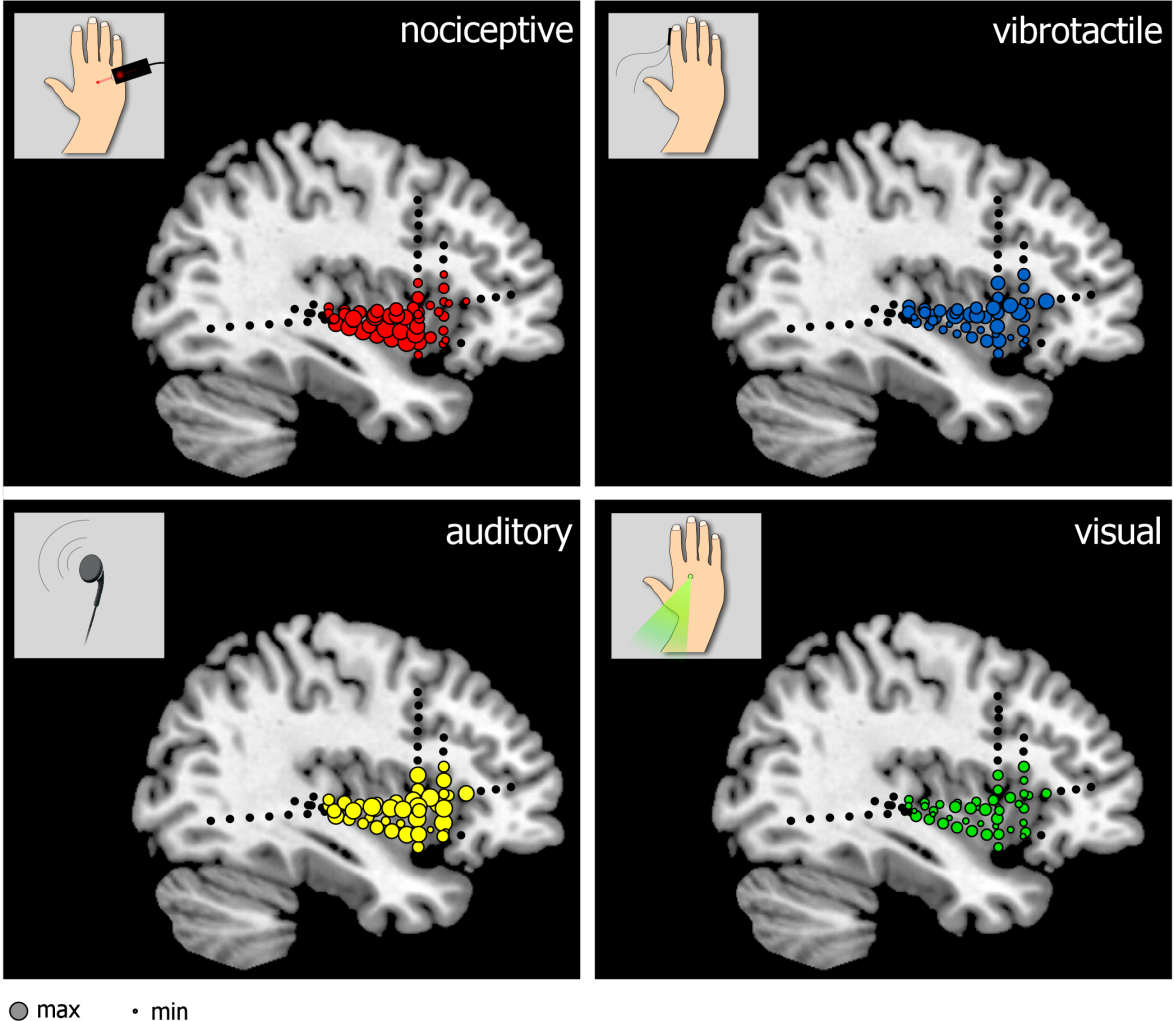
Spatial distribution of the amplitudes of LFPs elicited by nociceptive, vibrotactile, auditory, and visual stimuli across the different insular contacts (normalized across subjects). The size of each electrode contact represents the peak-to-peak amplitude of the biphasic wave elicited by each type of stimulus delivered to the contralateral side. doi:10.17605/OSF.IO/4R7PM.

For both peaks, there was no difference between the amplitude of the responses elicited by stimuli delivered to the ipsilateral and contralateral side (first peak: F = 1.02, *p* = .312; second peak: F = 0.52, *p* = .473). Furthermore, there was no difference between the amplitudes of the responses recorded from the anterior and posterior insula (first peak: F = 0.13, *p* = .723; second peak: F = 0.60, *p* = .441). The spatial distribution of the amplitudes of the LFPs elicited by the different types of stimuli modalities is shown in Fig 3.

### The Bulk of Nociceptive and Non-nociceptive Insular LFPs Originate from Spatially Indistinguishable Sources

Because the insula represents a relatively large area, and because it may contain spatially segregated subareas subtending different functions, it was crucial to determine whether the insular LFPs elicited by nociceptive stimulation and those elicited by non-nociceptive vibrotactile, auditory, and visual stimulation originate from spatially distinct or identical sources within the insula.

**Fig 4:**
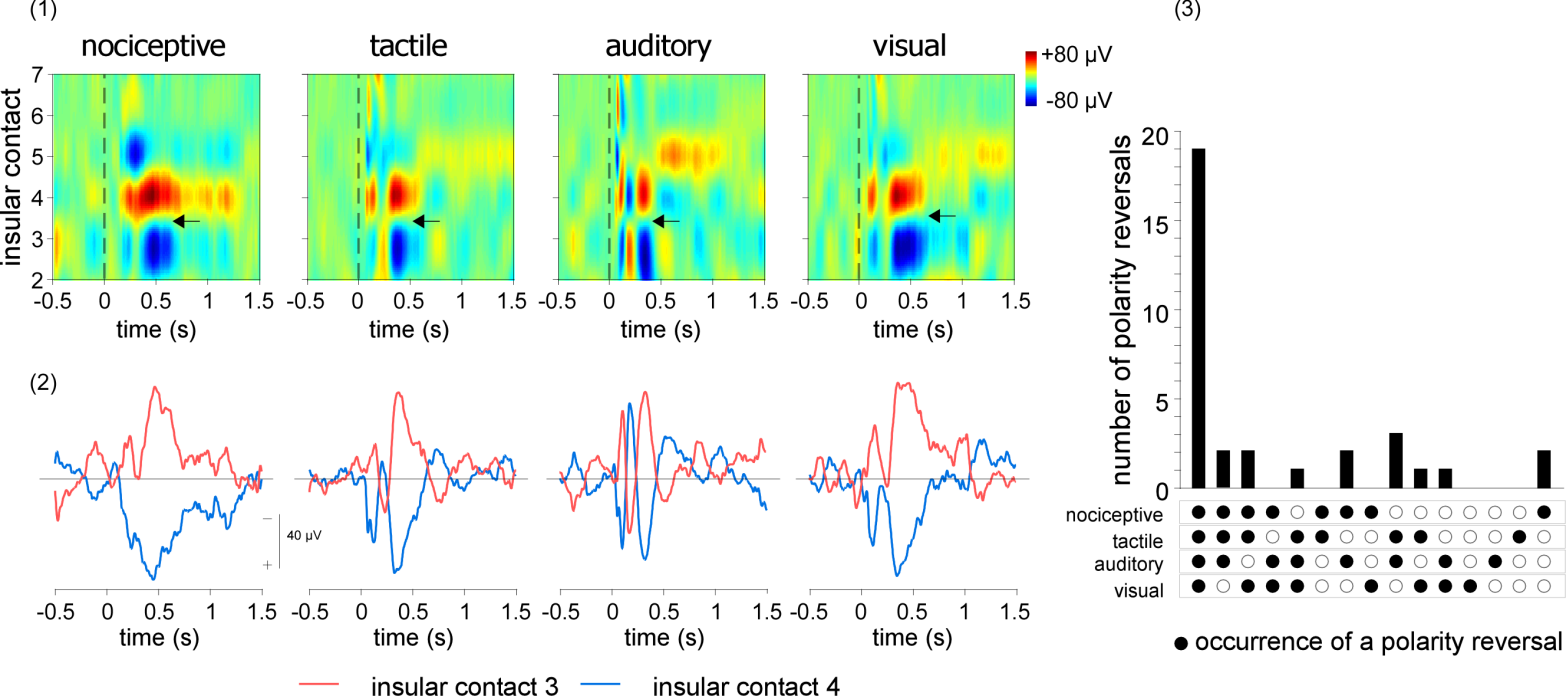
Linear CSD plots of the LFPs elicited by nociceptive, tactile, auditory, and visual stimuli delivered to the contralateral side. Upper left panel. CSD maps obtained from the right insula of a representative patient, expressing the recorded signals as a function of time (*x*-axis) and insular electrode contact location (*y*-axis). Note that polarity reversals are observed at the same insular locations for all four types of LFPs. One of these polarity reversals is shown by the horizontal arrows, between contacts 3 and 4. Lower left panel. CSD signals recorded from these two contacts. The signal shown for each insular contact corresponds to the signal measured from that contact, using the average of the two adjacent contacts as reference. Right panel. Total number of polarity reversals that occurred at the same contact locations across modalities and patients. In almost all cases, polarity reversals occurred at the same sites for all four modalities, indicating that, at least at the mesoscopic level of intracerebral EEG recordings, the locations of the sources generating nociceptive and non-nociceptive LFPs in the insula are largely identical. doi:10.17605/OSF.IO/4R7PM.

For this purpose, linear current source density (CSD) plots were computed by numerical differentiation to approximate the second order spatial derivative of the LFPs recorded across the different, evenly spaced contacts of each insular electrode [42]. The obtained signals were then used to compute two-dimensional maps expressing the recorded signals as a function of time and electrode contact location and to identify all electrode contact locations showing inversions of polarity (Fig 4, upper panel). At the mesoscopic level of intracerebral EEG recordings, the electrical activity generated in a given area can be summarized as an equivalent current dipole, located close to the center of activity, and having an orientation that is orthogonal to the activated cortical surface. Contacts showing an inversion of polarity may thus be considered as located closest to a source of activity, respectively in front and behind the dipole source.

In the vast majority of cases (Fig 4, lower panel), polarity reversals were observed at the same contacts for all four types of LFPs. This indicates that, at least at the level of intracerebral EEG recordings, the locations of the sources generating nociceptive LFPs in the insula can be considered as identical to the locations of the sources generating non-nociceptive vibrotactile, auditory, and visual elicited LFPs (Fig 4 and S2 Fig).

### Nociceptive and Non-nociceptive LFPs Elicited in the Insula Are Largely Explained by Multimodal Activity

Because the insula may be involved in multiple aspects of sensory processing, nociceptive and non-nociceptive LFP waveforms could reflect a combination of modality-specific and multimodal activities (i.e., unimodal neural activity specifically related to the processing of input belonging to a given sensory modality and multimodal neural activity reflecting higher-order processes that are independent of sensory modality). To test this hypothesis, we used a blind source separation algorithm based on a probabilistic independent component analysis (PICA) to break down the LFP waveforms elicited by all four types of stimuli and recorded at the different insular contacts into a set of independent components (ICs) [43]. When applied to multichannel electrophysiological recordings, this algorithm separates the recorded signals into a linear combination of ICs, each having a fixed spatial projection onto the electrode contacts and a maximally independent time course. Assuming that modality-specific and multimodal responses have nonidentical spatial distributions across insular contacts, PICA can be expected to separate these responses into distinct ICs.

The estimated number of independent sources contributing to the eight LFP waveforms (four modalities x two sides of stimulation) ranged, across insulae, between 2 and 6 (4.0 ± 1.5).

**Fig 5:**
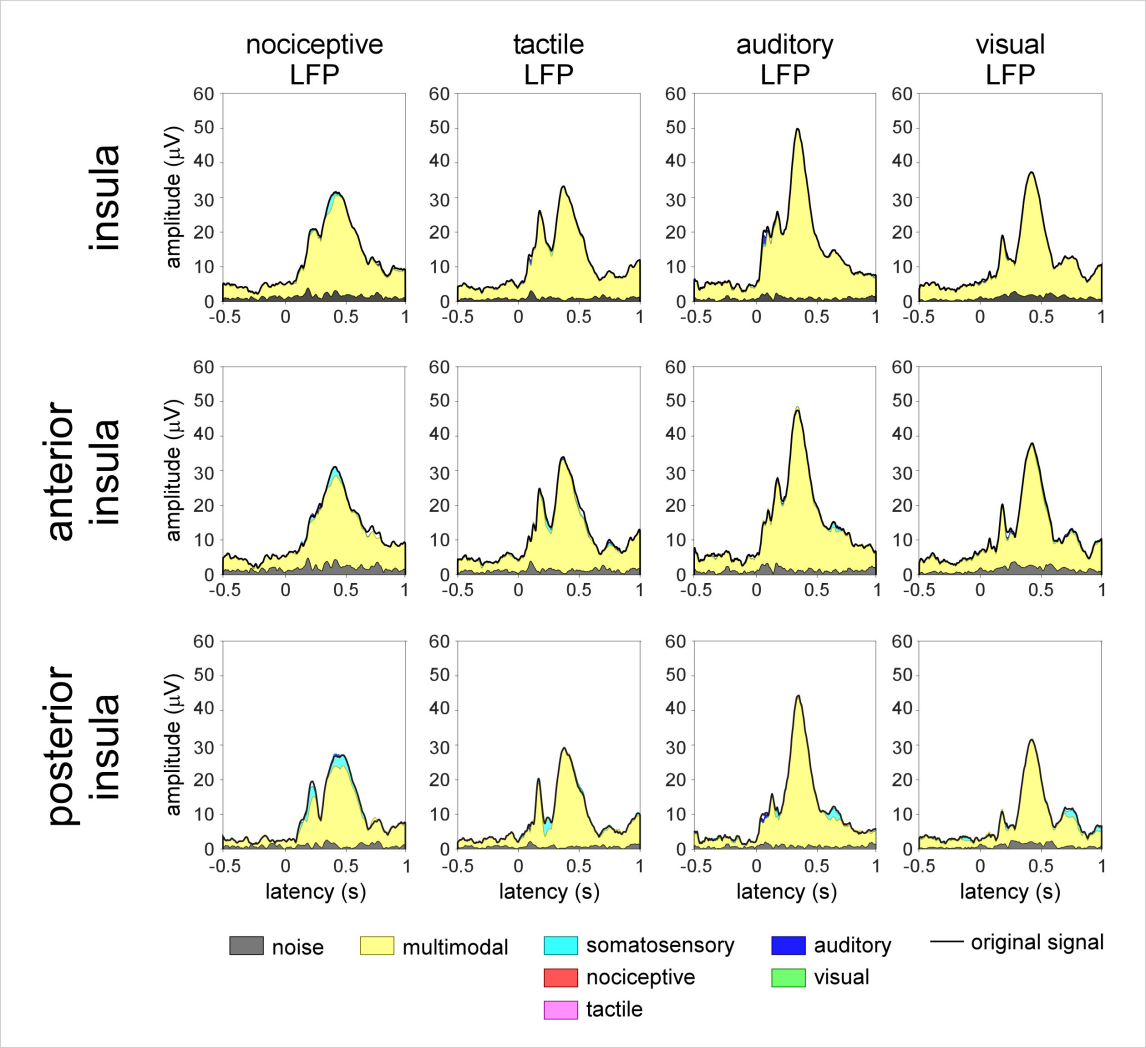
Blind source separation analysis of the LFPs recorded from the insula and elicited by nociceptive, tactile, auditory, and visual stimuli delivered to the contralateral hemibody. PICA was used to isolate the contribution of multimodal and modality-specific neural activities. The displayed waveforms correspond to the global field amplitude of the ICs, backprojected onto the electrode contacts, as a function of time. LFPs elicited by nociceptive and non-nociceptive stimuli (global field amplitude of the original signal: black waveform) can be almost entirely explained by multimodal sources of activity (yellow). A small amount of somatosensory-specific activity (cyan) also contributes to both the nociceptive and vibrotactile LFPs, in particular, those recorded from the posterior insula. Not a single nociceptive-specific component (red) is identified. doi:10.17605/OSF.IO/4R7PM.

Multimodal ICs (i.e., ICs contributing to the responses elicited by all four types of stimuli) were the main constituent of all LFPs, both when considering the responses elicited by stimuli to the contralateral side relative to the explored insula (3.0 ± 1.2 ICs; explaining 88% and 95% of the nociceptive LFP peaks, 98% and 93% of the vibrotactile LFP peaks, 95% and 95% of the auditory LFP peaks, and 74% and 78% of the visual LFP peaks; Fig 5) and when considering the responses elicited by stimulation of the ipsilateral side (S3 Fig). Taken together, this indicates that nociceptive and non-nociceptive LFPs recorded from the insula predominantly reflect the same source of multimodal cortical activity.

A smaller number of ICs appeared to contribute specifically to the LFPs elicited by somatosensory stimuli, regardless of whether the stimulus was nociceptive (Fig 5 and S3 Fig). In addition, a small number of ICs contributed specifically to the LFPs elicited by auditory stimuli. Most importantly, not a single IC was categorized as nociceptive specific.

## Discussion

Our results clearly show that, in both the anterior and posterior insula, LFPs generated by transient nociceptive stimuli are unspecific for nociception and the perception of pain. Indeed, the large biphasic response elicited by nociceptive stimuli at insular contacts was indistinguishable from the large biphasic responses elicited by non-nociceptive vibrotactile, auditory, and visual stimuli, apart from the expected differences in response latencies, which are easily explained by variations in the time required for stimulus transduction, as well as variations in the time required for the afferent volleys to reach the cortex. These responses were recorded from the anterior, medial, and posterior short gyri and from the anterior and posterior long gyri. Although none of our subjects presented contact locations in the superior portion of the anterior long gyrus, LFPs recorded in this region in response to nociceptive stimulation were shown to be identical in morphology to the responses in the other portions of the posterior insula [44].

Not only do we show that all stimuli elicit consistent LFPs in the posterior and anterior insula, we also show that the LFPs elicited by nociceptive and non-nociceptive stimuli originate from the same regions within the posterior and anterior insula. This is demonstrated by the fact that polarity reversals occur at the same electrode contact locations and by the fact that the LFPs elicited by all four types of stimuli have matching spatial distributions across insular contacts.

Finally, using a blind source separation algorithm, we show that the insular LFPs elicited by nociceptive stimuli can be largely explained by a source of activity also contributing to the LFPs elicited by non-nociceptive vibrotactile, auditory, and visual stimuli. This indicates that the recorded insular LFPs predominantly reflect a multimodal stage of sensory processing that is independent of nociception and the perception of pain.

These findings urge a reinterpretation of the evidence supporting a specific involvement of the insula in pain perception. Ostrowsky and collaborators [21] showed that direct electrical stimulation of the posterior insula can elicit an unpleasant somatic experience, involving shock, burning, or pricking sensations. However, they also observed that stimulation of the insula is equally likely to elicit nonpainful somatic sensations, such as paresthesiae and warm sensations. Furthermore, although vivid painful experiences have been reported following direct electrical stimulation of the insula, these seem to occur mainly when stimulating an epileptogenic area [45]. Similarly, although pain can be associated with epileptic activity in the insula, it remains an uncommon manifestation of insular epilepsy, which has only been observed in a few cases [3,4]. Finally, although studies have shown that lesions of the insula can impair the ability to perceive pain [5], there are also case reports of patients with extensive unilateral or bilateral insular damage showing little or no deficit in the ability to perceive pain, as indicated by the lack of changes in pain thresholds assessed using quantitative sensory testing [22,46].

At first glance, our results could appear to be in contradiction with the results of Frot et al. [47], showing that nonpainful stimuli do not elicit consistent LFPs in the posterior insula. It must be highlighted that the nonpainful stimuli used by Frot et al. [47] were low pulses of radiant heat eliciting a mild sensation of warmth. In contrast, the nonpainful stimuli used in the present study elicited a sensation whose perceived intensity was similar to the perceived intensity of nociceptive stimulation. Hence, the finding that weak thermal stimuli do not elicit LFPs in the posterior insula but more intense vibrotactile, auditory, and visual stimuli elicit consistent LFPs in the posterior insula could be primarily related to differences in stimulus salience (i.e., the property of a stimulus to “stand out” relative to neighboring stimuli).

Importantly, our finding that insular responses to transient sensory stimuli predominantly reflect multimodal activity is in agreement with several other studies suggesting a prominent role of the insula in cognition, attention, and human perception, independently of sensory modality [29,32,40,48–50]. The insula is a very heterogeneous region with a complex structural and functional connectivity. It is involved in a variety of functions, which are not limited to pain and nociception. Although it is often considered as a multidimensional integration site for pain [51], the insula is multisensory in nature. The insula is considered to be part of a frontoparietal control network commonly activated during tasks that require controlled information processing [52,53], as well as a core network [54–56] that is activated for the maintenance of focal attention. Furthermore, the insula has been related to the detection of salience [57], possibly constituting a hub connecting sensory areas to other networks involved in the processing and integration of external and internal information [49]. Such a multimodal salience network would allow gaining a coherent representation of different salient conditions, including, but not limited to, pain-related experiences [40,58]. This leads us to hypothesize that insular LFPs predominantly reflect multimodal activity involved in detecting, orienting attention towards, and reacting to the occurrence of salient sensory events, regardless of the sensory pathways through which these events are conveyed [59–61].

Alternative interpretations should be considered. Besides being involved in a number of sensory and cognitive processes, the insula has also been associated with autonomic function, interoception, and emotions. Patients with damage in the parietal opercular insular region show an impaired ability to recognize facial expressions of emotions and to experience empathy [62]. Moreover, insular activation has been associated with the experience of disgust and fear [63,64]. Craig [65,66] described the dorsal posterior insula as an interoceptive system that would give rise to distinct feelings that originate from inside the body, including pain, itch, temperature, sensual touch, muscular and visceral sensations, vasomotor activity, hunger, and thirst. By providing a sense of one’s own physical status, these feelings would reflect needs of the body and underlie mood and affective states. Furthermore, the insula could play an important role in generating autonomic responses, such as those triggered by the occurrence of a salient sensory stimulus [60] or those related to the autonomic expression of emotions [65]. Interestingly, these interpretations could also account for the recent finding that CBF in the posterior insula correlates with the varying magnitude of long-lasting ongoing pain [18].

Finally, one should be cautious to not overinterpret our results. Although our findings clearly question the notion that insular LFPs reflect processes specifically involved in the perception of pain, they do not exclude a specific involvement of the insula in pain perception. Unlike single unit recordings, LFPs sample the activity of neurons at the population level. Indeed, it is thought that the main contribution to LFPs derives from synchronous postsynaptic activity occurring in the apical dendrites of pyramidal neurons located in the cortex surrounding the electrode contact [67]. Therefore, one cannot exclude the possibility that LFPs elicited by nociceptive and non-nociceptive stimuli might reflect the activity of distinct neurons intermingled within the same subregions of the insula. However, single unit recordings performed in the monkey insula suggest that the population of truly nociceptive-specific neurons is extremely sparse [68].

In conclusion, by showing that, in the insula, LFPs elicited by nociceptive stimuli are spatially indistinguishable from the LFPs elicited by non-nociceptive vibrotactile, auditory, and visual stimuli, our results confute the widespread assumption that these brain responses constitute a signature for pain perception and its modulation. Does this constitute a demonstration that the insula cannot be considered as a “primary cortex for pain?” Although it is important to acknowledge the fact that the function of primary sensory cortices is probably not restricted to the processing of sensory input belonging to its corresponding sensory modality and, instead, that primary sensory cortices subsume multisensory integration functions [69–71], studies have shown that neurons sensitive to other modalities are rare within primary visual, auditory, and somatosensory areas. For this reason, and in striking contrast with our insular recordings, large-amplitude LFPs are recorded in primary sensory areas only if the eliciting stimulus activates afferents belonging to the corresponding sensory modality [72,73]. Therefore, although our results clearly do not exclude the existence of nociceptive-specific or pain-specific processes in the insula, they do highlight the lack of a spatially segregated parcel of the human insula that could be considered as a “primary cortex” for pain.

## Methods

All experimental procedures were approved by the local Research Ethics Committee (B403201316436) and were performed in compliance with the Code of Ethics of the World Medical Association (Declaration of Helsinki). All participants gave written informed consent.

### Participants

Six patients (three females, mean age: 27, range 19–43 y) recruited at the Department of Neurology of the Saint Luc University Hospital (Brussels, Belgium) were included in the study. All participants suffered from focal epilepsy and, before functional surgery, were investigated using depth electrodes implanted in various brain regions suspected to be the origin of the seizures, including the anterior and posterior insula. The intracerebral EEG was recorded from a total of 72 sites. The localization of the insular electrodes for each patient can be seen in Fig 2 and S1 Fig. None of the patients presented ictal discharge onset in the insula, and low voltage fast activity was never present in this area during spontaneous seizures.

### Procedure

The study was conducted at the patient bedside. Before the beginning of the experiment, the procedure was explained to the participant, who was exposed to a small number of test stimuli for familiarization. The experiment consisted of two sessions of four blocks each, one session per side of stimulation. In each block, the subject received stimuli belonging to one of four sensory modalities: nociceptive, vibrotactile, auditory, and visual. Each stimulation block consisted of 40 stimuli. The order of the stimulation blocks was randomized across participants. A blocked design was chosen to ensure that expecting the possible occurrence of a nociceptive stimulus would not affect the responses elicited by non-nociceptive stimuli [74]. The interstimulus interval (ISI) was large, variable, and self-paced by the experimenter (5–10 s). Participants were instructed to keep their gaze fixed on a black cross (3 x 3 cm) placed in front of them on the edge of the bed, at a distance of ~2 m, 30° below eye level, for the whole duration of each block. To ensure that each stimulus was perceived and to maintain vigilance across time, participants were asked to press a button as soon as they felt the stimulation. Furthermore, participants provided a subjective intensity rating for each stimulus on a scale ranging from 0 to 10 (0 was defined as “undetected” and 10 was defined as “maximum intensity”). At the end of each block, the patients were asked to report whether they had perceived any of the stimuli as painful.

### Sensory Stimuli

Nociceptive somatosensory stimuli consisted of 50-ms pulses of radiant heat generated by a CO_2_ laser (wavelength: 10.6 μm). The laser beam was transmitted via an optic fiber, and focusing lenses were used to set the diameter of the beam at the target site to 6 mm. The laser stimulator was equipped with a radiometer providing a continuous measure of the target skin temperature, which was used in a feedback loop to regulate laser power output. The power output of the laser was adjusted to raise the target skin temperature to 62.5°C in 10 ms and to maintain this temperature for 40 ms. To prevent nociceptor fatigue or sensitization, the laser beam was manually displaced after each stimulus [75]. Each laser stimulus elicited a clear painful pinprick sensation, previously shown to be related to the activation of Aδ fiber skin nociceptors [74]. Non-nociceptive somatosensory stimuli consisted in a 50-ms vibration at 250 Hz, delivered via a recoil-type vibrotactile transducer driven by a standard audio amplifier (Haptuator, Tactile Labs, Canada) and positioned on the palmar side of the index fingertip. Auditory stimuli were loud, lateralized sounds (0.5 left/right amplitude ratio) delivered through earphones. The sounds consisted in a 50-ms tone at 800 Hz. Visual stimuli were 50-ms punctate flashes of light delivered by means of a light-emitting diode (LED) with a 12 lm luminous flux, a 5.10 cd luminous intensity, and a 120° visual angle (GM5BW97333A, Sharp, Japan), placed on the hand dorsum.

### Intracerebral EEG Recordings and Analysis

For each patient, a tailored implantation strategy was planned on the basis of the regions considered most likely to be ictal onset sites or propagation sites. The desired targets, including the insular cortex, were reached using commercially available depth electrodes (AdTech, Racine, Wisconsin, United States; contact length: 2.4 mm; contact spacing: 5 mm), implanted using a frameless stereotactic technique through burr holes. The placement was guided by neuronavigation based on a 3D T1-weighted MRI sequence. A post-implantation 3D-T1 (3D-T1W) MRI sequence was used to accurately identify single contact localizations.

Intracerebral EEG recordings were performed using a Deltamed (Paris, France) acquisition system. Additional bipolar channels were used to record electromyographic (EMG) and electrocardiographic (EKG) activity. All signals were acquired at a 512 Hz sampling rate and analyzed offline using Letswave 6 [76].

The continuous recordings were referenced to the average of the two mastoid electrodes (A1A2), segmented into 1.5-s epochs (−0.5 to 1.0 s relative to stimulus onset) and band-pass filtered (0.3–40 Hz). After baseline subtraction (reference interval: −0.5 to 0 s relative to stimulus onset), separate average waveforms were computed for each subject, stimulus type (nociceptive somatosensory, non-nociceptive somatosensory, auditory, and visual), and side of stimulation. For two of the subjects, trials containing strong artifacts were corrected using an independent component analysis (ICA) algorithm [77] or removed after visual inspection.

The latencies and amplitudes of the LFPs were compared using a LMM analysis as implemented in IBM SPSS Statistics 22 (Armonk, New York: IBM) with “modality” (four levels: nociceptive, vibrotactile, auditory, and visual), “contact location” (two levels: anterior and posterior insula) and stimulation “side” (two levels: stimuli delivered to the ipsilateral or contralateral side relative to the explored insula) as fixed factors. Assuming that the responses recorded from the different contacts of a given subject are not independent, “subject” was used as a contextual variable grouping the insular contacts. Parameters were estimated using restricted maximum likelihood (REML) [78]. In all analyses, main effects were compared using the Bonferroni confidence interval adjustment.

### Linear CSD Plots

Linear CSD plots were computed by numerical differentiation to approximate the second order spatial derivative of the LFPs recorded across the different, evenly spaced contacts of the insular electrode [42]. The obtained signals were then used to compute two-dimensional maps expressing the recorded signals as a function of time and electrode contact location, using spline interpolation. The spatiotemporal maps were then used to identify visually all electrode contact locations showing polarity reversal, as well as to compare the spatial distribution of the LFPs elicited by nociceptive and non-nociceptive stimuli.

### PICA

A blind source separation algorithm was used to isolate the contribution of multimodal and modality-specific neural activities to the LFPs elicited by nociceptive and non-nociceptive vibrotactile, auditory, and visual stimuli. For each participant, the blind source separation was performed using runica [77,79], an automated form of the Extended Infomax ICA algorithm [80]. When applied to multichannel recordings, this algorithm separates the recorded signal into a linear combination of ICs, each having a fixed spatial projection onto the electrode contacts and a maximally independent time course. When ICA is unconstrained, the total number of ICs equals the total number of channels. If the number of ICs is far greater than the actual number of independent sources, ICs containing spurious activity will appear because of overfitting. On the other hand, if the number of ICs is much smaller than the actual number of sources, information will be lost because of underfitting. For this purpose, ICA was constrained to an effective estimate of the intrinsic dimensionality of the original data (PICA) [81]. The estimate was obtained using a method based on maximum likelihoods and operating on the eigenvalues of a principle component analysis [43].

For each participant, the algorithm was applied to the eight average waveforms (4 types of stimuli x 2 sides) obtained at all insular contacts (8–12 contacts).

To estimate the contribution of each obtained IC to the LFPs elicited by the different types of stimuli, the time course of the amplitude of each IC (μV) was expressed as the standard deviation from the mean (z-scores) of the prestimulus intervals of all eight waveforms (−0.5 to 0 s). If the poststimulus amplitude of an IC was greater than z = 1.5, the IC was considered as reflecting stimulus-evoked activity. Each of these ICs was then classified according to its contribution to the eight LFP waveforms. For each IC and each side of stimulation, we computed the ratio between the z-score of a specific modality and the z-scores of the other three modalities [40,34]. If the ratio was ≥2 for one stimulus modality versus each of the other three modalities, the IC was classified as modality specific (i.e., nociceptive, non-nociceptive vibrotactile, auditory, or visual). If the computed ratio was ≥2 for both nociceptive and non-nociceptive somatosensory stimuli versus auditory and visual stimuli, the IC was classified as somatosensory specific. Finally, ICs that contributed to all four LFP waveforms were classified as multimodal. Crucially, the obtained results were not critically dependent on the number of dimensions used to constrain ICA or on the arbitrarily defined threshold of z ≥ 2. In fact, all ICs were unambiguously multimodal or modality specific, and IC classifications obtained using other cut-off values ranging between 1.5 and 3.5 yielded identical results.

### Anatomical Electrode Contact Localization

The anterior insula was identified as the region encompassing the short insular gyri (anterior, middle, and posterior), the pole of the insula, and the transverse insular gyrus. The posterior insula was identified as the region composed of the anterior and posterior long insular gyri [38]. Individual MRI were normalized to a standard echo-planar imaging (EPI) template in MNI space, using Statistical Parametric Mapping (SPM8, Wellcome Department of Imaging Neuroscience, London, United Kingdom). The anatomical location of each contact was identified on the 3D-T1W sequence with the help of multiplanar reformations, by a neuroradiologist (MMS) with 10 y of experience.

## Supporting Information

S1 Fig LFPs recorded from the anterior and posterior insula of five patients, following nociceptive, tactile, auditory, and visual stimulation. All stimuli were delivered contralateral to the explored insula. The recordings obtained in the other two patients are shown in Fig 2. doi:10.17605/OSF.IO/4R7PM.
(TIF)

S2 Fig Linear CSD plots of the LFPs elicited by nociceptive, tactile, auditory, and visual stimuli delivered to the contralateral side relative to the explored insula. For each participant and stimulation type, CSD maps were obtained by expressing the recorded signals as a function of time (*x*-axis) and insular electrode contact location (*y*-axis). Note that polarity reversals are observed, in the majority of cases, at the same insular locations for all four types of LFPs, indicating that the locations of the sources generating nociceptive and non-nociceptive LFPs in the insula are largely identical. For each subject, an example of polarity reversal identified in the four modalities is shown by horizontal arrows, together with the relative CSD signal. The signal measured at a given insular contact is displayed using the average of the two adjacent contacts. The same approach was used to generated the CSD plots obtained in one representative patient shown in Fig 3. doi:10.17605/OSF.IO/4R7PM.
(TIF)

S3 Fig Blind source separation analysis of the LFPs recorded from the insula and elicited by nociceptive, tactile, auditory, and visual stimuli delivered to the ipsilateral hemibody (ipsilateral hand or ipsilateral side relative to the explored insula). The displayed waveforms correspond to the global field amplitude of the ICs as a function of time. Like the LFPs elicited by stimuli delivered to the contralateral side, the LFPs are almost entirely explained by a large contribution of multimodal activity (yellow). A small distinct contribution of somatosensory-specific activity (cyan) also contributes to both the nociceptive and vibrotactile LFPs, in particular, those recorded from the posterior insula. Not a single nociceptive-specific component (red) is identified. doi:10.17605/OSF.IO/4R7PM.
(TIF)

## Abbreviations

CBF: cerebral blood flow
CSD: current source density
EEG: electroencephalography
EKG: electrocardiogram
EMG: electromyogram
EPI: echo-planar imaging
fMRI: functional magnetic resonance imaging
IC: independent component
ICA: independent component analysis
ISI: interstimulus interval
LED: light-emitting diode
LFP: local field potential
LMM: linear mixed models
MRI: magnetic resonance imaging
PET: positron emission tomography
PICA: probabilistic independent component analysis
REML: restricted maximum likelihood
SMCS: Plateforme technologique de Support en Methodologie et Calcul Statistique
