# Supplementary figures and images for: Nociceptive Local Field Potentials Recorded from the Human Insula Are Not Specific for Nociception
Source: PLoS Biol. 2016 Jan 6;14(1):e1002345. doi: 10.1371/journal.pbio.1002345 (PMC4703221; doi:10.1371/journal.pbio.1002345)

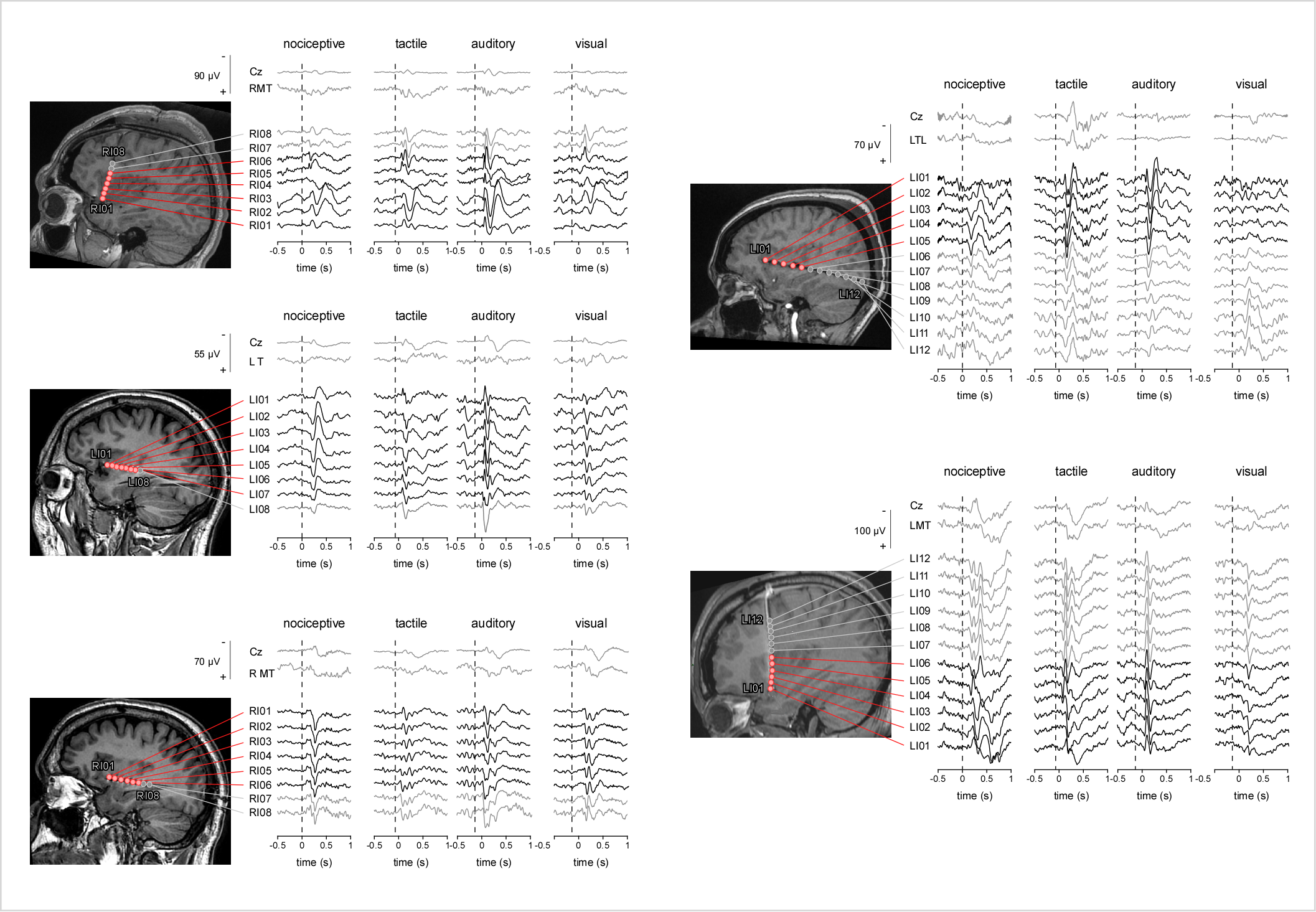

Supplement: S1 Fig — All stimuli were delivered contralateral to the explored insula. The recordings obtained in the other two patients are shown in Fig 2. doi:10.17605/OSF.IO/4R7PM. (TIF) [file pbio.1002345.s001.tif]

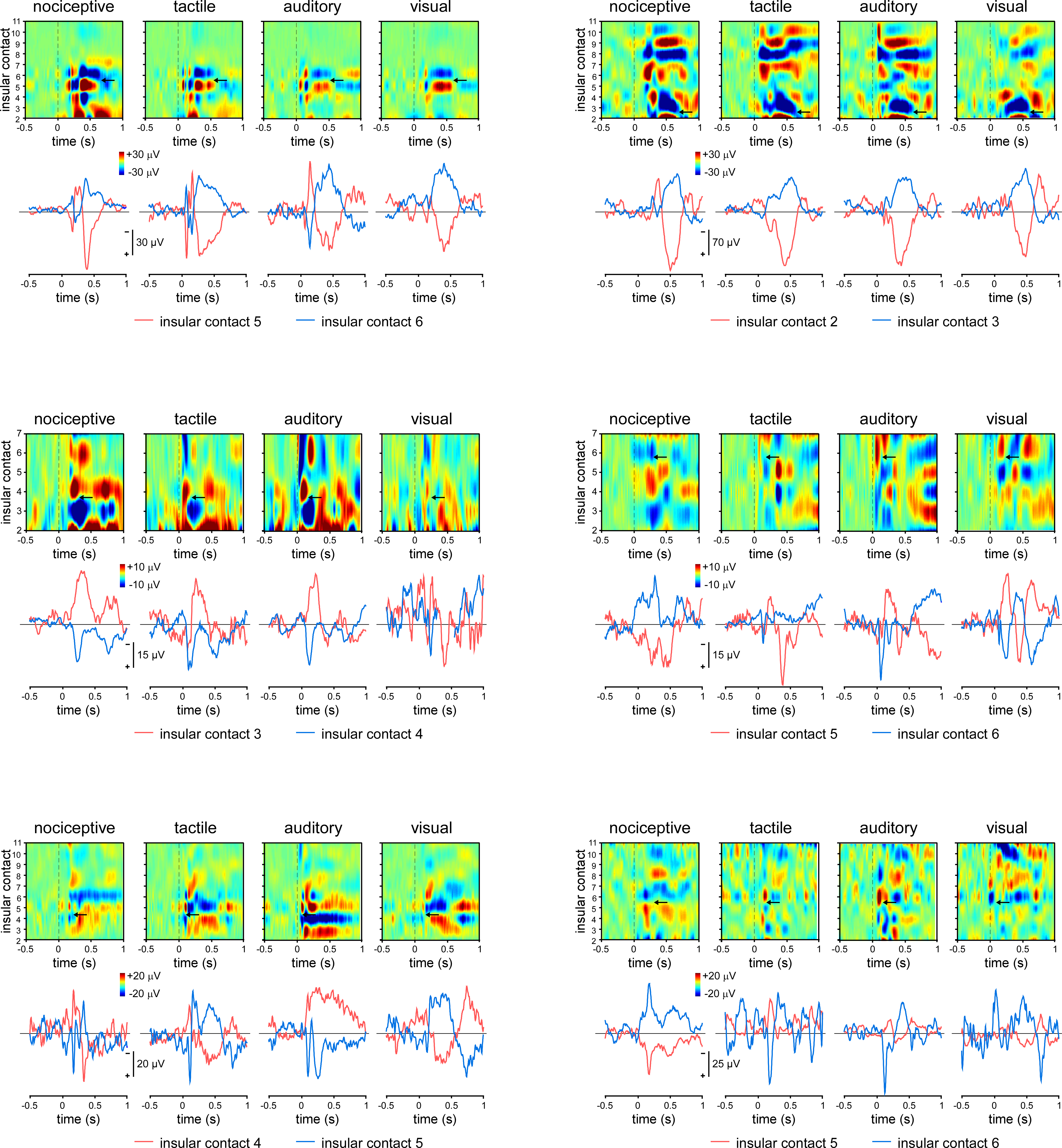

Supplement: S2 Fig — For each participant and stimulation type, CSD maps were obtained by expressing the recorded signals as a function of time (x-axis) and insular electrode contact location (y-axis). Note that polarity reversals are observed, in the majority of cases, at the same insular locations for all four types of LFPs, indicating that the locations of the sources generating nociceptive and non-nociceptive LFPs in the insula are largely identical. For each subject, an example of polarity reversal identified in the four modalities is shown by horizontal arrows, together with the relative CSD signal. The signal measured at a given insular contact is displayed using the average of the two adjacent contacts. The same approach was used to generated the CSD plots obtained in one representative patient shown in Fig 3. doi:10.17605/OSF.IO/4R7PM. (TIF) [file pbio.1002345.s002.tif]

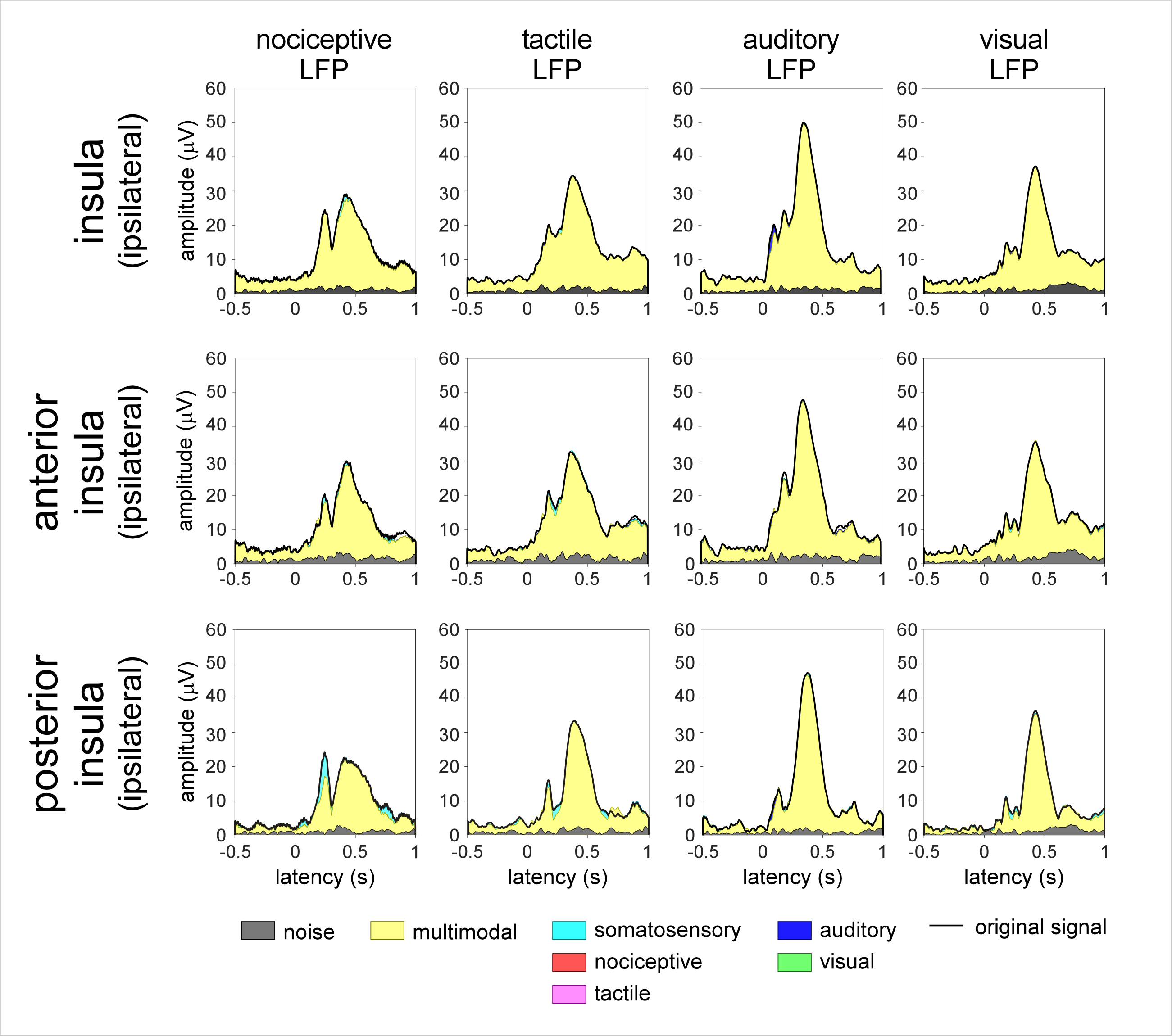

Supplement: S3 Fig — The displayed waveforms correspond to the global field amplitude of the ICs as a function of time. Like the LFPs elicited by stimuli delivered to the contralateral side, the LFPs are almost entirely explained by a large contribution of multimodal activity (yellow). A small distinct contribution of somatosensory-specific activity (cyan) also contributes to both the nociceptive and vibrotactile LFPs, in particular, those recorded from the posterior insula. Not a single nociceptive-specific component (red) is identified. doi:10.17605/OSF.IO/4R7PM. (TIF) [file pbio.1002345.s003.tif]
